# Diversification of *Schistosoma japonicum* in Mainland China Revealed by Mitochondrial DNA

**DOI:** 10.1371/journal.pntd.0001503

**Published:** 2012-02-14

**Authors:** Qin Ping Zhao, Ming Sen Jiang, Hui Fen Dong, Pin Nie

**Affiliations:** 1 Department of Parasitology, School of Basic Medical Science, Wuhan University, Wuhan, Hubei Province, China; 2 State Key Laboratory of Freshwater Ecology and Biotechnology, Institute of Hydrobiology, Chinese Academy of Sciences, Wuhan, Hubei Province, China; University of Melbourne, Australia

## Abstract

**Background:**

*Schistosoma japonicum* still causes severe parasitic disease in mainland China, but mainly in areas along the Yangtze River. However, the genetic diversity in populations of *S. japonicum* has not been well understood across its geographical distribution, and such data may provide insights into the epidemiology and possible control strategies for schistosomiasis.

**Methodology/Principal Findings:**

In this study infected *Oncomelania* snails were collected from areas in the middle and lower (ML) reaches of the Yangtze River, including Hubei, Hunan, Anhui, Jiangxi and Jiangsu provinces, and in the upper reaches of the river, including Sichuan and Yunnan provinces in southwest (SW) China. The adult parasites obtained from experimentally infected mice using isolated cercariae were sequenced individually for several fragments of mitochondrial regions, including Cytb-ND4L-ND4, 16S-12S and ND1. Populations in the ML reaches exhibited a relatively high level of diversity in nucleotides and haplotypes, whereas a low level was observed for populations in the SW, using either each single fragment or the combined sequence of the three fragments. Pairwise analyses of *F*-statistics (*F*st) revealed a significant genetic difference between populations in the ML reaches and those in the SW, with limited gene flow and no shared haplotypes in between. It is rather obvious that genetic diversity in the populations of *S. japonicum* was significantly correlated with the geographical distance, and the geographical separation/isolation was considered to be the major factor accounting for the observed difference between populations in the ML reaches and those in the SW in China.

**Conclusions:**

*S. japonicum* in mainland China exhibits a high degree of genetic diversity, with a similar pattern of genetic diversity as observed in the intermediate host snails in the same region in China.

## Author Summary

Despite the existing threat of schistosomiasis in some rural areas along the Yangtze River, the genetic diversity of *Schistosoma japonicum* has not been investigated across its wide geographical distribution in China, and such information may provide insight into the disease epidemiology and the development of its control measures. In this study, the adult parasites, obtained through infecting mice with cercariae from snails of the genus *Oncomelania* collected from a wide range of localities in currently endemic areas of schistosomiasis in the middle and lower (ML) reaches of the Yangtze River, and in Sichuan and Yunnan provinces in the upper reaches of the river in southwest (SW) China, were sequenced individually for mitochondrial genes. In general, a relatively high degree of genetic variation was observed in populations in the ML reaches in terms of nucleotide and haplotype diversity, but a low level was observed in populations in the SW. The significant difference in genetic diversity as revealed by *F*-statistics, and the existence of no shared haplotypes, were observed between populations in the ML reaches and those in the SW, indicating the effect of geographical separation/isolation upon the schistosomes and probably the parasite-snail system in China.

## Introduction

Schistosomiasis is one of the most neglected tropical diseases, with six species in the *Schistosoma* still infecting more than 200 million people in the world [1]–[3]. Schistosomiasis japonica is distributed in Indonesia, Philippines, and China. In mainland China, this parasitic disease is the most severe zoonosis infecting about 360,000 people and about 1% buffalo and/or cattle in endemic regions, particularly in lake/marshland and hilly areas of Hubei, Hunan, Anhui, Jiangxi and Jiangsu provinces and mountainous areas of Sichuan and Yunnan provinces [4]. Over the last 50 years, continuous efforts involving various measures, such as health education, snail control, community chemotherapy and environmental management have contributed significantly to the dramatic reduction in infection levels and epidemic areas of this parasitic disease in China, setting China as one of the most successful countries in control of schistosomiasis in the world [5]–[8]. However, recently available data have suggested that schistosomiasis has re-emerged over the last decade, probably as a severe threat once again to human health especially in rural areas of mainland China [9], [10]. The drastic pathogenesis, the number of reservoir hosts involved in epidemiology and the vast endemic areas of schistosomiasis japonica have inevitably resulted in a less investigated status for *S. japonicum* in respect with its genetic diversity, host immune response etc. when compared with other schistosomes [6], [11], [12].

The genus *Oncomelania*, which is the intermediate host of *S. japonicum*, was classified into different species and/or subspecies according to their morphology, biogeography and phylogeny [13], [14]. With the distinct diversity of snails in the genus *Oncomelania* which has been verified using various markers [14]–[16], the diversity of the parasite *S. japonicum* is of great interest for research from a co-evolutionary point of view. How diverse the digenean *S. japonicum* really is in such a large geographical range has not been well assessed especially in mainland China. An accurate measure of its population genetic diversity is certainly needed to clarify our understanding on the epidemiology of schistosomiasis [17], which may be also useful for implementing control measures, and for developing drugs or potential vaccines, as worms of different genetic backgrounds may respond differently to such treatments [18], [19].

In recent years, several molecular markers have been used to detect the variability of *S. japonicum* populations. Gasser et al. [20] found the variability among 7 geographical isolates across mainland China using the random amplified polymorphism DNA (RAPD) technique and suggested a potential strain complex for *S. japonicum*. Sorensen et al. [21] reported differences between *S. japonicum* populations from 6 localities in mainland China using NADH dehydrogenase subunit 1 (ND1) gene, but could not detect variability conclusively at the intrapopulation level. Bøgh et al. [22] did find 15 types of ND1 conformations and 23 types of cytochrome c oxidase subunit 1 (CO1) conformations in 9 populations from 7 provinces across mainland China by single-strand conformational polymorphism (SSCP). These results did in fact suggest the significant polymorphism among *S. japonicum* in mainland China, but provided very limited information relating to the population genetic diversity of this species. Upon the identification of polymorphic microsatellite loci, Shrivastava et al. [6] investigated the genetic variation of *S. japonicum* populations from 8 geographical locations in 7 endemic provinces across mainland China, and a high level of polymorphism was reported between and within populations. They considered that populations of *S. japonicum* in mainland China could be separated mainly into the populations in Sichuan and Yunnan provinces as being in southwest (SW) China and those in low-lying lake regions along the middle and lower (ML) reaches of Yangtze River. With three partial mitochondrial genes (cox3, nad4 and nad5) from 28 individual adult worms, Zhao et al. [23] reported recently that all parasites from SW China were grouped together, whereas those from the ML reaches of Yangtze River were not clustered together. However, the reports by Shrivastava et al. [6] and Zhao et al. [23] both contained limited specimens from relatively few localities, which may not represent the geographical distribution of this schistosome, and thus not its exact population genetic diversity, in mainland China. A comprehensive analysis is therefore needed using more molecular markers to examine more populations of *S. japonicum* from a wide range of its geographical distributions, especially in severely endemic areas along the ML reaches of Yangtze River in China.

In this study, mitochondrial DNA sequences including Cytb-ND4L-ND4, 16S-12S and ND1 were examined for *S. japonicum* collected from localities in seven provinces of China, where schistosomiasis is geographically endemic. The diversity in nucleotides and haplotypes was analyzed for different populations based on each of the three mitochondrial sequences and their combined sequences. Phylogenetic tree and parsimony network were constructed for observed haplotypes, and the genetic distance was examined against the geographical distance in order to understand the genetic diversity in populations of *S. japonicum* in mainland China.

## Materials and Methods

### Ethics statement

The procedures involving animals were carried out in accordance with the guidelines of the Association for Assessment and Accreditation of Laboratory Animal Care International (AAALAC). The animal study protocol was approved by the Institutional Animal Care and Use Committee of Wuhan University.

### Collection of parasite specimens

**Figure 1:**
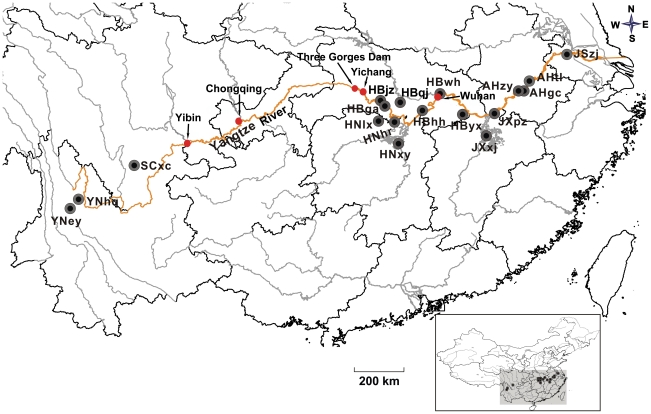
A schematic map showing sample localities in endemic areas of schistosomiasis in mainland China. A total of 18 localities were indicated as black solid circles, where *Schistosoma japonicum* infected snails were collected. The names of sample localities were abbreviated as the same as in Table 1. Red solid circles indicating cities and the Three-Gorge Dam, respectively.

The intermediate host, *Oncomelania hupensis*, from 18 localities of 7 schistosomiasis endemic provinces in mainland China, including Hubei, Hunan, Anhui, Jiangxi, Jiangsu provinces in the ML reaches of Yangtze River, and Sichuan and Yunnan provinces which are in the higher reaches of the river in SW China, but separated from the ML reaches by mountain ranges (Fig. 1 and Table 1), were collected and transported to laboratory from October 2005 to October 2006. After one month captivity, snails were washed and exposed individually in water for 3 h in a vial under light at 25°C to stimulate the emergence of cercariae for identifying the *S. japonicum* infection. Overall, snails from different localities had an infection rate ranging from 0.1% to 4%. To generate adult worms, the best source of DNA, 10 field-collected infected snails from each locality, with the exception of Zongyang in Anhui province (AHzy) and Pengze in Jiangxi province (JXpz) where only three and four infected snails were obtained respectively, were exposed to light for 4 hours to stimulate the emergence of cercariae. Five laboratory mice were infected percutaneously with 30 cercariae per mouse for each geographical locality. 6 weeks following the infection, adult worms were retrieved by perfusion from mesenteric veins using 0.9% NaCl, and worms from each mouse infected with cercariae were pooled together, and washed extensively in saline before being preserved in 95% ethanol at 4°C.

**Table 1:** Sample localities in relation with haplotypes for *Schistosoma japonicum* obtained in the present study.

Locality*	Province	Latitude; longitude	Haplotype†
Wuhan (HBwh)	Hubei	30°38′N; 114°20′E	H1, 2, 3, 22, 23, 25, 48, 66
Jinzhou (HBjz)	Hubei	30°20′N; 112°02′E	H5, 6, 43, 55, 56, 61, 74, 80
Qianjiang (HBqj)	Hubei	30°17′N; 112°47′E	H5, 7, 24, 44, 45, 75
Yangxin (HByx)	Hubei	29°49′N; 115°13′E	H4, 20, 21, 41, 46, 52, 77, 94
Honghu (HBhh)	Hubei	29°58′N; 113°39′E	H8, 9, 10
Gong'an (HBga)	Hubei	30°09′N; 112°10′E	H11, 34, 78
Lixian (HNlx)	Hunan	29°32′N; 111°57′E	H47, 50, 53, 59, 62, 95
Xiangyin (HNxy)	Hunan	28°41′N; 112°43′E	H49, 53, 69, 86, 87, 90, 96
Huarong (HNhr)	Hunan	29°31′N; 112°33′E	H12, 14, 36, 50, 51, 63, 64, 65, 66, 83
Tongling (AHtl)	Anhui	31°06′N; 117°50′E	H35, 60, 75, 85
Guichi (AHgc)	Anhui	30°45′N; 117°37′E	H13, 42, 76, 84
Zongyang (AHzy)	Anhui	30°44′N; 117°25′E	H81, 82, 84
Xinjian (JXxj)	Jiangxi	28°59′N; 116°09′E	H37, 39, 79, 88, 89, 91, 92, 93
Pengze (JXpz)	Jiangxi	29°52′N; 116°28′E	H15, 16, 17, 18, 19, 71, 72, 73
Zhenjiang (JSzj)	Jiangsu	32°10′N; 119°18′E	H38, 40, 54, 57, 58, 67, 68, 70
Xichang (SCxc)	Sichuan	27°49′N; 102°22′E	H29, 30, 31, 32
Eryuan (YNey)	Yunnan	26°09′N; 99°52′E	H26, 27, 28, 33
Heqing (YNhq)	Yunnan	26°30′N; 100°12′E	H27, 33

*The locality is listed as the city or county where the intermediate host snail *Oncomelania hupensis* infected with *Schistosoma japonicum* was collected, and each locality is designed with a two-letter province code followed by two-letter city or county code.

**†:** Haplotypes were deduced from combined mitochondrial data set, containing Cytb-ND4L-ND4, ND1 and 16S-12S regions.

### DNA extraction, PCR amplification, and sequencing of mitochondrial genes

The total genomic DNA was extracted individually from both male and female schistosomes using a standard sodium dodecyl sulfate-proteinase K procedure [24]. Each worm was incubated and thawed in 200 µl extraction buffer containing 50 mM Tris-HCl, 50 mM EDTA, 100 mM NaCl, 1% SDS and 100 µg/ml proteinase K, at 56°C for 2 h with gentle mixing. DNA in solution was extracted using standard phenol/chloroform purification, followed by 3 M sodium acetate (pH 5.2) and ethanol precipitation. Pellets of DNA were washed in 70% ethanol, air-dried, and resuspended in 10 µl TE (pH 8.0).

For each adult worm, three fragments, i.e. Cytb-ND4L-ND4, ND1 and 16S-12S of the mitochondrial genome were sequenced. For the Cytb-ND4L-ND4 fragment, the forward primer ND4F (5′- TTGGGGGTTGTCATGCGGAGTA -3′) and the reverse primer ND4R (5′- CAAATACCCAATAGCAACGGAACAC -3′) were used based on available GenBank sequence AF215860. For the ND1 fragment, the forward primer ND1F (5′- TAGAGGGTTTGTTGGTTGTTTTG -3′) and the reverse primer ND1R (5′- ACCATACTTTCATACTACTGCC -3′) were used based on available GenBank sequence AF215860. For the 16–12S fragment, the forward primer 16S-12SF (5′- GATTATTTCTAGTTCCCGAATGG -3′) and the reverse primer 16–12SR (5′- TGTAACGCACAACAACCTATACC -3′) were used based on available GenBank sequence AF215860. The PCR protocols were 94°C for 3 min followed by 30 cycles of 94°C for 30 s, 58°C (for ND1) or 63°C (for Cytb-ND4L-ND4 and 16S-12S) for 30 s, and 72°C for 90 s and then a final elongation step at 72°C for 10 min. The amplified products were purified on 1.0% agarose gel stained with ethidium bromide, using the DNA gel extraction kit (Omega Bio-Tek). The purified PCR products were sequenced using ABI PRISM BigDye Terminators v3.0 Cycle Sequencing (ABI). The DNA sequences were deposited in the GenBank database under accession numbers FJ851893–FJ852573.

### Sequence alignments and analyses

Sequences were aligned using ClustalX1.83 [25] at default settings followed by manual correction in SEAVIEW [26] for each molecular marker. DNAsp version 4.0 [27] was used to define the haplotype. The three parts, i.e. Cytb-ND4L-ND4, ND1 and 16S-12S, of mitochondrial data were also combined and aligned into a new combined mitochondrial data set, with this combined sequence named as combined mtDNA.

Nucleotide divergences within and between populations were calculated in Arlequin3.11 [28] and DNAsp. Genetic variation within different populations was estimated by calculating nucleotide diversity (π) and haplotype diversity (*h*) values. Selective neutrality was tested with Tajima's *D* [29] and Fu's *F* test [30]. The pairwise genetic difference was estimated for all populations by calculating Wright's *F*-statistics (*F*st) based on gene flow (Nm). A Mantel g-test to compare the correlation between pairwise distance and geographical distance among localities was analyzed in Arlequin, with geographic distances (km) for the correlation analysis between geographical distance and genetic distance calculated using the great circle distance between localities.

The phylogenetic analysis for 96 haplotypes generated using combined mitochondrial DNA data was performed with Bayesian inference (BI), which was carried out with MrBayes 3.1 [31] under the best-fit substitution model. Analyses were run for 1×10^6^ generations with random starting tree, and four Markov chains (with default heating values) sampled every 100 generations. Posterior probability values were estimated by generating a 50% majority rule consensus tree following the discard of first 3000 trees as part of a burn-in procedure. The HKY+I+G model was determined as the best-fit model of sequence evolution by using the hierarchical likelihood ratio tests implemented in Modeltest 3.7 [32]. The phylogenetic tree was rooted using *Schistosoma mansoni* as outgroup.

The genetic structure was phylogenetically evaluated by constructing unrooted parsimony network of haplotypes for combined mtDNA data sets using TCS version 1.21 [33].

## Results

### Diversity within and among populations based on three separate mitochondrial DNA sequences

The primary sequence data were obtained by amplifying and sequencing three partial regions of the mitochondrial genome, i.e. Cytb-ND4L-ND4 with 793–794 bp, ND1 with 767 bp, and 16S-12S with 1463–1466 bp. Measures of diversity of haplotypes and nucleotides within populations on the basis of the three mitochondrial regions are presented in Tables S1, S2 and S3, respectively. The highest values for the diversity were all observed for populations in the ML reaches, and the lowest all in populations from the SW (for details regarding each fragment, see Tables S1, S2 and S3). The pairwise genetic distance among all 18 populations showed a high degree of variation, as revealed respectively from the three different mitochondrial regions (for details, see Tables S4, S5 and S6).

**Figure 2:**
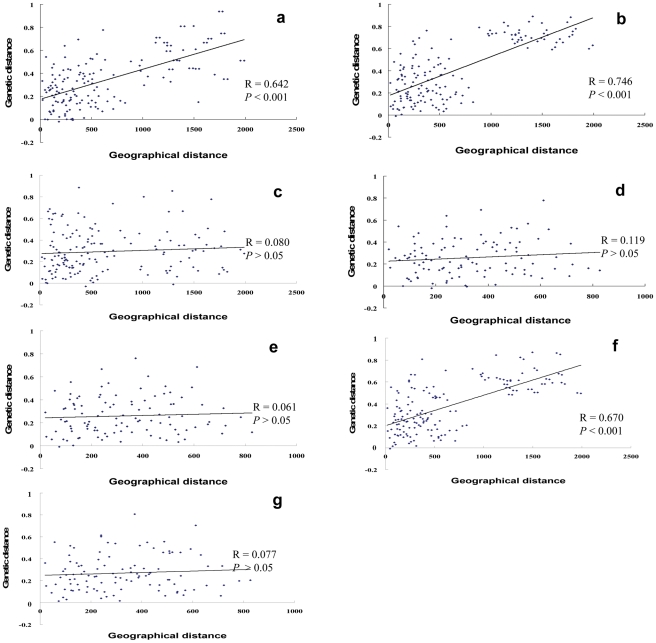
Scatter plots of geographical distance against genetic distance for *Schistosoma japonicum* in mainland China. A, B, and C showing the relationship from all 18 populations based on Cytb-ND4L-ND4, 16S-12S and ND1 fragments, respectively; d and e showing the relationship from 15 populations along the ML reaches based on Cytb-ND4L-ND4 and 16S-12S, respectively, with the exclusion of three populations from Sichuan and Yunnan provinces; F and G showing the relationship based on combined mitochondrial DNA sequences of Cytb-ND4L-ND4, ND1 and 16S-12S from all 18 populations and 15 populations along the ML reaches, respectively.

A significant correlation was observed between geographical distance and genetic distance (pairwise *F*st) for all 18 populations for Cytb-ND4L-ND4 (R = 0.642, *P*<0.001) and 16S-12S (R = 0.746, *P*<0.001), respectively, which indicates that genetic distance increased with the increase in geographical distance (Fig. 2a, b). No significant correlation was detected when ND1 was used, with the correlation coefficient R = 0.080 (*P*>0.05) (Fig. 2c). However, among 15 populations in the ML reaches, the value of the correlation coefficient decreased to 0.119 (*P*>0.05) and 0.061 (*P*>0.05) for Cytb-ND4L-ND4 and 16S-12S, respectively (Fig. 2d, e), implying that the genetic distance was not correlated with the geographical distance for populations in the ML reaches of Yangtze River.

Although some base substitutions were observed, selective neutrality of the observed nucleotide polymorphisms was suggested for *S. japonicum*, as indicated either by Tajima's *D* or Fu's *F* test (*P*>0.05) in each of the three regions.

### Diversity within and between populations based on combined mitochondrial DNA sequences

As many studies have shown that longer genes contain generally more variable characters with proportionally more signals, and hence yield accurate phylogenetic estimates than shorter ones [34]–[36], the combined mitochondrial data sets were then deduced from 169 specimens by aligning combined Cytb-ND4L-ND4, ND1 and 16S-12S sequences (combined mtDNA), which had a range of 3024 to 3027 bp, resulted in 3028 characters, including gaps, and 166 variable sites (113 parsimony informative sites). A total of 96 mitochondrial haplotypes was observed (Table 1). Measures of haplotype and nucleotide diversity based on combined mtDNA are presented in Table 2. The highest values in the diversity of haplotype and nucleotide were all observed for populations in the ML reaches, and the lowest were all in populations from the SW, which is consistent with the findings from the three separate mitochondrial DNA sequences. 88 haplotypes were isolated from 143 specimens in five provinces along the ML reaches, with the mean haplotype and nucleotide diversity being 0.987±0.003 and 0.0036±0.0001, respectively. However, only 8 haplotypes were isolated from 26 specimens in the SW, with the haplotype and nucleotide diversity being 0.766±0.075 and 0.0017±0.0003, respectively. The *F*st of all pairwise analyses varied from 0.482 to 0.870 between populations in the ML reaches and those in the SW (Table 3), showing highly significant difference (*P*<0.001). Among the 3 populations in the SW, the *F*st between SCxc and two Yunnan populations (YNey and YNhq) showed highly significant differences (*P*<0.001), whereas no significant difference was observed between YNey and YNhq (*P*>0.05). Among the 15 populations in the ML reaches, the *F*st varied from 0.014 to 0.807 (Table 3), with most of them being significantly different (*P*<0.05). When all specimens were classified into two populations according to whether they were from above or below the three Gorges region, i.e. population in the ML reaches of the Yangtze River and population in Sichuan and Yunnan provinces of the SW China, the value of genetic distance (*F*st) and the gene flow (Nm) between them was 0.381 (*P*<0.001) and 0.410, respectively.

**Table 2:** Within-locality diversity in combined mitochondrial DNA sequences of *Schistosoma japonicum* §.

Locality	n	μ	*h*	π
HBwh	9	8	0.972±0.064	0.0038±0.0005
HBjz	9	7	0.972±0.064	0.0030±0.0004
HBqj	10	6	0.911±0.062	0.0030±0.0003
HByx	10	8	0.956±0.059	0.0034±0.0004
HBhh	10	3	0.600±0.131	0.0008±0.0002
HBga	10	3	0.644±0.101	0.0031±0.0004
HNlx	10	6	0.867±0.085	0.0020±0.0004
HNxy	9	7	0.944±0.070	0.0029±0.0004
HNhr	10	10	1.000±0.045	0.0030±0.0004
AHtl	9	4	0.750±0.112	0.0018±0.0003
AHgc	7	4	0.714±0.181	0.0024±0.0009
AHzy	10	3	0.600±0.131	0.0008±0.0002
JXxj	10	8	0.956±0.059	0.0035±0.0004
JXpz	10	8	0.956±0.059	0.0030±0.0004
JSzj	10	8	0.956±0.059	0.0031±0.0004
SCxc	7	4	0.810±0.130	0.0013±0.0002
YNey	10	4	0.711±0.117	0.0008±0.0003
YNhq	9	2	0.389±0.164	0.0006±0.0003
All	169	96	0.986±0.003	0.0038±0.0001

**§:** The combined sequences contain those of Cytb-ND4L-ND4, ND1 and 16S-12S. n, the number of worms sequenced; μ, the number of unique haplotypes within a locality; *h,* haplotype diversity ± standard deviation; π, nucleotide diversity ± standard deviation.

**Table 3:** Geographical population pairwise genetic distance (*F*st) and gene flow (Nm) based on combined mt DNA sequences§.

	HBwh	HBjz	HBqj	HByx	HBhh	HBga	HNlx	HNxy	HNhr	AHtl	AHgc	AHzy	JXxj	JXpz	JSzj	SCxc	YNey	YNhq
**HBwh**		4.133	3.834	3.923	1.123	1.625	1.143	1.662	3.544	1.908	1.704	0.425	0.976	1.590	1.892	0.306	0.437	0.438
**HBjz**	0.108		34.322	29.400	0.977	2.801	1.870	4.701	17.114	6.841	4.527	0.586	1.437	2.570	2.626	0.269	0.420	0.413
**HBqj**	0.115	0.014		12.375	1.248	2.513	1.266	3.004	10.037	7.508	4.098	0.505	1.303	2.054	2.672	0.284	0.462	0.456
**HByx**	0.113	0.017	0.039		1.010	2.268	1.567	3.661	8.972	7.434	4.628	0.581	1.438	2.426	2.594	0.312	0.493	0.491
**HBhh**	0.307*	0.338*	0.286*	0.331*		0.878	0.313	0.464	0.838	0.409	0.460	0.119	0.413	0.698	0.524	0.091	0.119	0.107
**HBga**	0.235*	0.151	0.166	0.181	0.362*		0.656	1.422	1.666	1.543	2.459	0.560	1.103	1.546	0.997	0.235	0.322	0.319
**HNlx**	0.304*	0.210*	0.283*	0.241*	0.615*	0.432*		1.819	4.175	1.004	0.650	0.210	0.594	0.605	1.933	0.195	0.296	0.284
**HNxy**	0.231*	0.096	0.143	0.120	0.518*	0.260*	0.216		3.640	3.803	3.975	0.594	1.699	1.339	2.028	0.250	0.367	0.361
**HNhr**	0.124	0.028	0.047	0.053	0.373*	0.230*	0.107	0.121		5.173	1.890	0.399	1.085	1.398	7.545	0.308	0.537	0.530
**AHtl**	0.207*	0.068	0.062	0.063	0.549*	0.244*	0.332*	0.116	0.088		5.114	0.407	1.386	1.522	2.062	0.206	0.359	0.341
**AHgc**	0.226*	0.099	0.109	0.098	0.520*	0.169	0.434*	0.112	0.209*	0.089		0.879	2.233	2.544	1.124	0.188	0.251	0.240
**AHzy**	0.540*	0.460*	0.497*	0.462*	0.807*	0.471*	0.704*	0.457*	0.555*	0.551*	0.362*		0.771	0.502	0.329	0.074	0.087	0.078
**JXxj**	0.338*	0.258*	0.277*	0.257*	0.547*	0.311*	0.457*	0.227*	0.315*	0.265*	0.182*	0.393*		1.482	1.132	0.312	0.360	0.360
**JXpz**	0.239*	0.163	0.195*	0.171	0.417*	0.244	0.452*	0.271*	0.263*	0.247*	0.164	0.499*	0.252*		0.977	0.235	0.301	0.297
**JSzj**	0.209*	0.159*	0.157*	0.161*	0.488*	0.333*	0.205*	0.197*	0.062	0.195*	0.307*	0.603*	0.306*	0.338*		0.346	0.507	0.501
**SCxc**	0.621*	0.650*	0.638*	0.616*	0.845*	0.680*	0.719*	0.667*	0.618*	0.707*	0.726*	0.870*	0.615*	0.679*	0.591*		0.252	0.248
**YNey**	0.533*	0.544*	0.520*	0.503*	0.807*	0.608*	0.628*	0.576*	0.482*	0.582*	0.665*	0.851*	0.581*	0.624*	0.496*	0.664*		∞
**YNhq**	0.533*	0.548*	0.523*	0.504*	0.824*	0.610*	0.637*	0.580*	0.485*	0.594*	0.675	0.864*	0.581*	0.627*	0.499*	0.668*	−0.009	

**§:** The combined sequences contain those of Cytb-ND4L-ND4, ND1 and 16S-12S. The names of sample localities were abbreviated as the same as in Table 1.

*F*st values in lower matrix. Nm values in upper matrix.

*indicating *P*<0.05.

Significant correlation was also observed between geographical distance and genetic distance (pairwise *F*st) among all 18 populations for combined mtDNA (R = 0.670, *P*<0.001), indicating that genetic distance increased with the increase in geographical distance (Fig. 2f). Among 15 populations in the ML reaches, the value of the correlation coefficient decreased to 0.077 (*P*>0.05) (Fig. 2g), implying that the genetic distance was not correlated with the geographical distance for populations in the ML reaches of Yangtze River.

### Phylogenetic relationship based on combined mitochondrial DNA sequences

**Figure 3:**
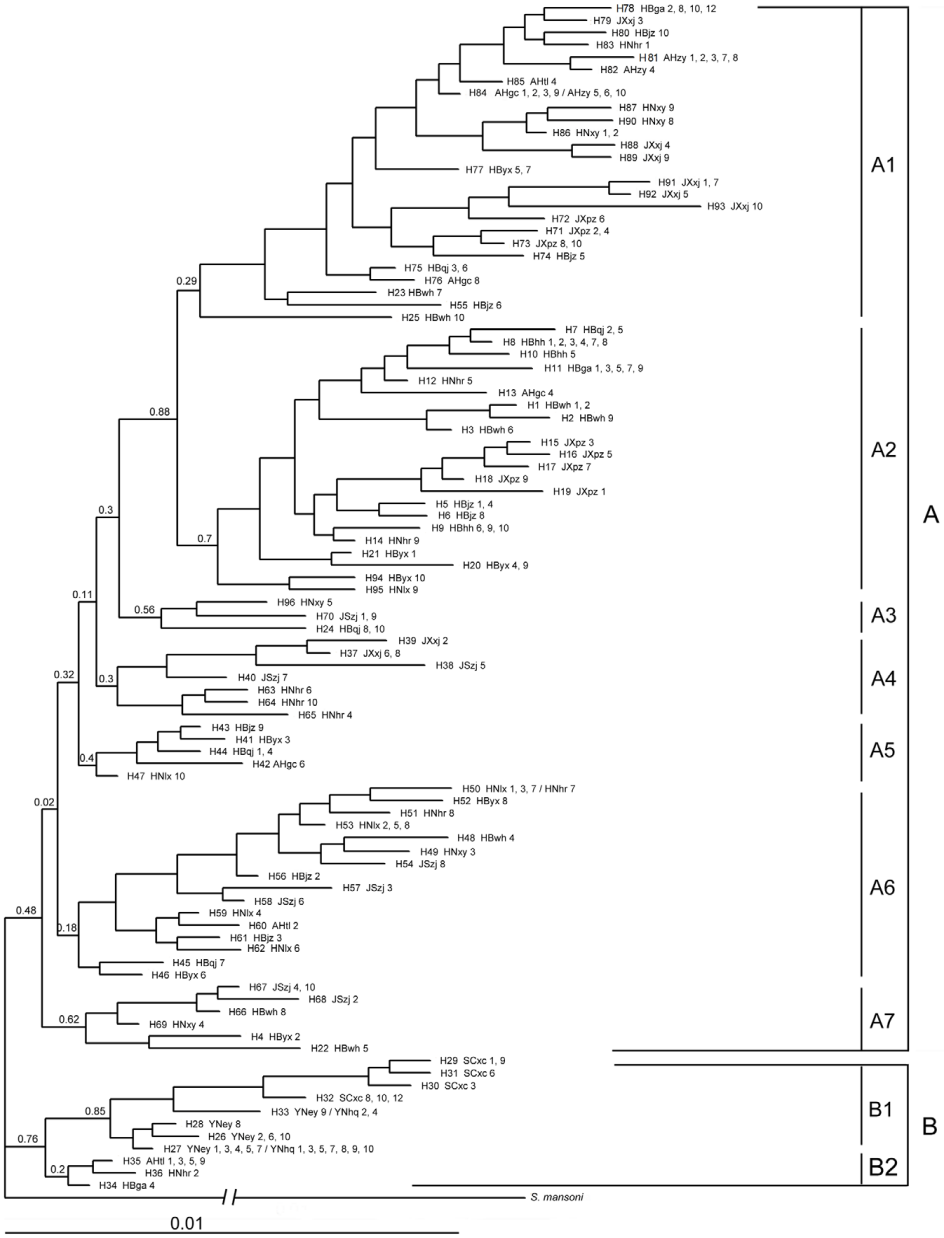
Bayesian inference tree based on combined mitochondrial DNA sequences for *Schistosoma japonicum* in mainland China. The combined sequences contain those of Cytb-ND4L-ND4, ND1 and 16S-12S. The letter H with the number after each branch represents different haplotypes, followed by sample locality as abbreviated in Table 1, and the serial number of isolated individuals which shared the same haplotype. The branch leading to the outgroup *S. mansoni* was shortened for a better presentation of the tree.

As shown in the Bayesian phylogenetic tree (Fig. 3), two clades can be clearly separated. Clade A contains almost all haplotypes from all five provinces in the ML reaches of the Yangtze River. Although various divergence and some subclades were observed within this clade, support probabilities for each clade were generally very low. Haplotypes in the ML reaches were clustered in various subclades, and no obvious lineage was observed for haplotypes from different provinces along the ML reaches. However, subclades A1 and A2 include most haplotypes from Hubei, Hunan, Anhui, and Jiangxi provinces, and subclade A6 includes haplotypes from Hubei, Hunan, Anhui, and Jiangsu provinces. It is apparent that clade B can be separated into two distinct subclades, B1 and B2, with clade B1 having a high support probability and containing only haplotypes from Sichuan and Yunnan provinces in SW China, and B2 containing three haplotypes from three provinces in the ML reaches. Surprisingly, other trees (NJ, ML, MP; not shown), although inconsistent in their respects, all had such two clades containing haplotypes from SW China, and three from the ML reaches, despite a relatively low level of support probabilities.

### Haplotype network based on combined mitochondrial DNA sequences

**Figure 4:**
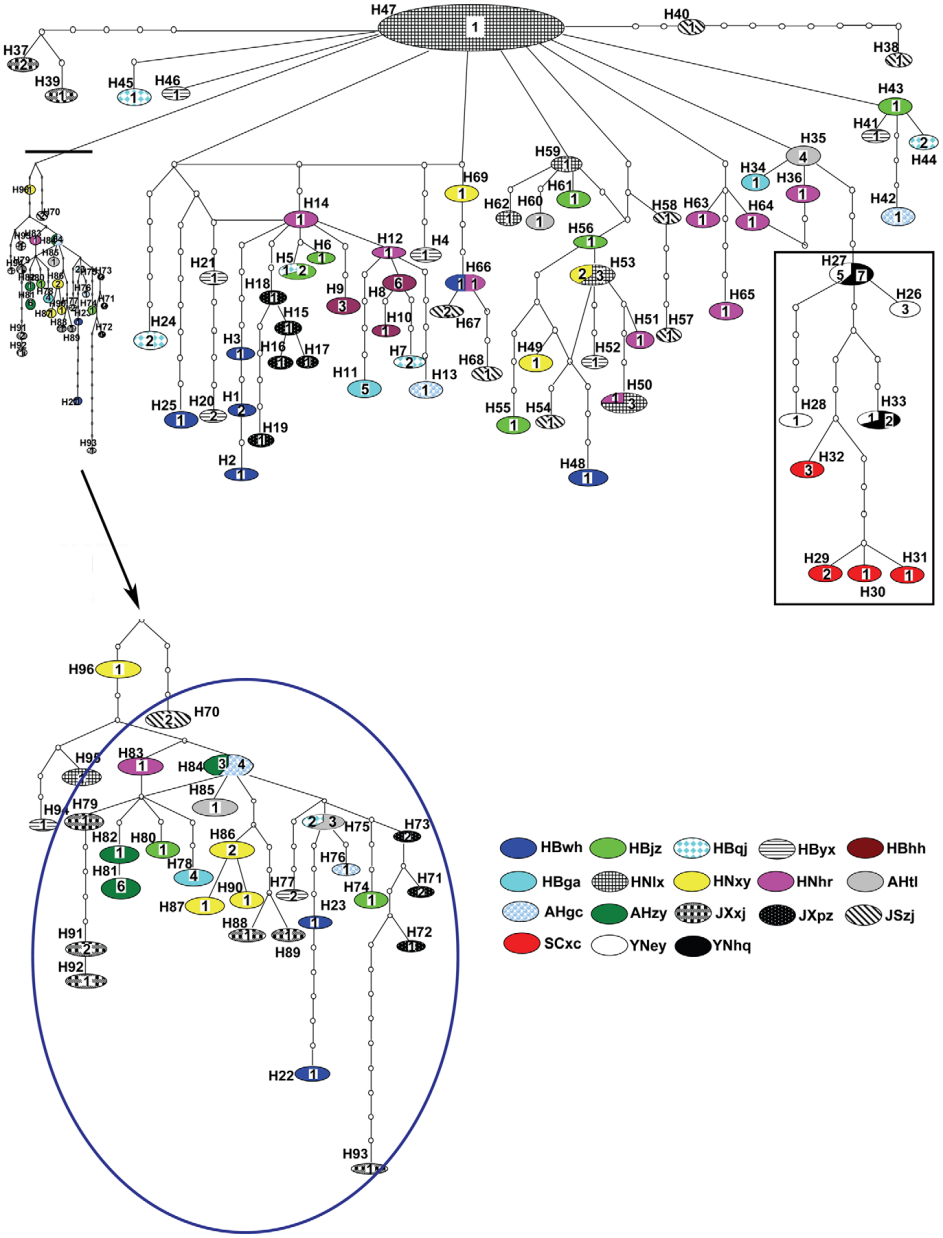
Unrooted parsimony network of combined mitochondrial DNA sequences haplotypes for *Schistosoma japonicum* in mainland China. The combined sequences contain those of Cytb-ND4L-ND4, ND1 and 16S-12S. Ovals indicate sampled haplotypes, which have designated numbers beside them. Numbers inside ovals indicate those individuals which share the same haplotype from different geographical populations. Small empty circles indicate un-sampled or extinct haplotypes. Each connection represents one mutational step. Rectangular box refers correspondingly to clade B1 in Fig. 3. The arrow indicates the expanded clade under the dark line, and the clade marked in blue circle contains most haplotypes (from H71 to H93) within clade A1 in Fig. 3.

The network constructed by statistical parsimony from 96 haplotypes on the basis of combined mtDNA sequences showed some characters as observed in the phylogenetic tree. The haplotype network was rather complicated, without any obvious lineages for those haplotypes from localities in the ML reaches (Fig. 4). However, all haplotypes from SW (from H26 to H33) were clustered together (Fig. 4), which corresponded exactly to clade B1 in Fig. 3, and this clade contained no haplotypes from the ML reaches of Yangtze River, but was related with a few haplotypes from the ML reaches (Fig. 4), as also indicated in clade B2 which formed, together with B1, into clade B (Fig. 3). A relatively large network containing haplotypes (from H71 to H93) from about 10 localities (Fig. 4) showed some similarity with clade A1 in Fig. 3, in composition of haplotypes. It is, however, impossible to detect any other patterns of haplotype networks, and impossible to find other geographical relationships or characteristic lineages in other network branches, which is largely consistent with the complex structure of clade A in Fig. 3.

## Discussion

The difference in genetic diversity of *S. japonicum* populations was demonstrated in samples collected from currently epidemic areas of schistosomiasis in mainland China, with the use of three mitochondrial fragments, Cytb-ND4L-ND4, ND1 and 16S-12S, respectively, and the combined sequences of these three fragments. The present study contains the mostly widespread and the largest number of *S. japonicum* populations in any attempts so far to examine the parasite genetic diversity in China. Overall, populations of *S. japonicum* in mainland China showed a relatively large degree of variation in terms of nucleotide and haplotype diversity. However, it is apparent that across the geographical distribution of schistosomiasis endemic areas in China, the genetic distance was correlated significantly with geographical distance when Cytb-ND4L-ND4, 16S-12S, and combined mtDNA were used, although non-significance was observed for ND1. It is even more obvious that as revealed through analyses of nucleotide and haplotype diversity, populations in Hubei, Hunan, Anhui, Jiangxi and Jiangsu provinces, namely in the ML reaches of Yangtze River showed a much larger degree of genetic variation than those in Sichuan and Yunnan provinces of the SW China in the upper reaches of the river, and no haplotypes were shared between populations in the ML reaches and those in the SW. Significant difference was also observed in genetic distance between populations in the ML reaches and populations in the SW, as revealed in pairwise analyses using individual and/or combined mitochondrial sequences.

Along the Yangtze River, are the endemic areas of schistosomiasis, and severe epidemic areas are mainly in the ML reaches [5]. However, in the Three Gorges area that is from Yichang going upwards to Yibin (Fig. 1), human schistosomiasis has never been reported [10]. It is quite obvious that the distribution of *S. japonicum* is geographically separated by the gorge area of the river. This apparent geographical separation may account for the observed difference in no-shared haplotypes, and in the genetic distance for *S. japonicum* between areas in the ML reaches of Yangtze River and areas in the SW China. When populations from the ML reaches and from the SW were further grouped separately, the *F*st value (0.381) was greater than 0.25, a value which was considered to be ‘very great’ by Wright [37] for genetic differentiation between populations. It is therefore all indicated that a large level of genetic differentiation has evolutionarily occurred for *S. japonicum*, due to at least the geographical separation by the Three Gorges area and mountains. Phylogenetic analyses and haplotype network may support this conclusion, as parasites from Sichuan and Yunnan provinces in the SW were all closely clustered in the phylogenetic tree and the haplotype network. Using different molecular markers, other authors [6], [23] have also, to some extent, detected the genetic difference between *S. japonicum* populations in the SW and those in the flood plain of the ML reaches of the Yangtze River.

Despite the finding that the mean nucleotide and haplotype diversity of populations in the SW were rather low when compared with the same parameters in the ML reaches, the genetic distance had some significant difference between the population from Sichuan, SCxc, and the two populations from Yunnan, YNey and YNhq, as revealed by *F*st of pairwise analyses using ND1, 16S-12S, and the combined mtDNA sequences, with the exception of Cytb-ND4L-ND4. Sichuan and Yunnan provinces are both distributed in Hengduan Mountains, and schistosomiasis was reported historically in various localities in these two provinces [38]. As various mountain ranges and rivers, as well as intermountain basins, are the general features in Hengduan Mountains [39], there must be some degree of geographical isolation in the distribution of *S. japonicum* in this region at a large geographical scale. However, only three populations were included in the present study and efforts to obtain more parasite samples have been unsuccessful, although the intermediate host snails were collected in a much wider range (unpublished data), due possibly to the continuous and extensive practices in either snail control or human chemotherapy in the two provinces. Thus, whether there is an effect of geographical isolation on populations of *S. japonicum* in this mountainous area will likely remain unknown, and whether the observed low level of genetic variation in these populations resulted from a recent bottleneck effect as a consequence of intensive control practices may also remain to be answered.

Ecological habitats were thought to affect population genetic diversity of *S. japonicum* in mainland China [40]. The mountainous habitats in Sichuan and Yunnan provinces may differ obviously from the habitats for the intermediate host in the ML reaches, in several aspects such as in hydrology, altitude and soil etc. [41], [42], but the difference should mostly be attributed to the geographical separation, rather than a simple impact from habitat difference. In the ML reaches of Yangtze River, it was impossible to clarify any patterns of haplotype clustering in relation to types of sample localities or to provinces, as haplotypes from a single locality were generally clustered in different clades. It can thus be speculated that *S. japonicum* might have experienced frequent gene flows in most populations in this region (Table 3). The localities for *O. hupensis* in the ML reaches have extensive physical connections through channels with the Yangtze River. With frequent occurrence of floods in the Yangtze River basin, especially in its ML reaches, snails in these habitats can be dispersed and subsequently deposited widely in various localities, and this naturally occurred instance was, in a previous research, proposed to explain the high genetic diversity of *O. hupensis* in the ML reaches [16]. It was further considered that this distinct genetic diversity in snail intermediate hosts may have strong implications in genetic diversity of schistosomes in mainland China [16], as demonstrated clearly in the present study. The accumulation of mixed sources of snails, especially infected snails can reconstitute the parasite population, leading to the existence of various haplotypes within a single population, and also to the limited degree of genetic distance between populations in the ML reaches as observed in the present study, which supports the speculation by Davis et al. [43] that floods may be the cause of the widespread mixing and dispersal of snails, leading to greater genetic diversity in *O. hupensis* populations along the Yangtze River plains compared with populations in SW China.

Surprisingly, the number of haplotypes, being 80 and 13 for the intermediate host snails in the ML reaches, and in Sichuan and Yunnan provinces [16], matches roughly, if not coincidently, with the number of haplotypes, 88 and 8, for *S. japonicum* in the ML reaches and in Sichuan and Yunnan provinces in this study, respectively. The intermediate host snails and the schistosome in China exhibit a lesser degree of genetic diversity in the SW, but a relatively larger degree in the ML reaches of the Yangtze River, as reported in a previous study on the intermediate host snails [16] and in this study. No shared haplotypes were observed either in the intermediate host snails or in the schistosomes between localities from the ML reaches and from the SW. Zhao et al. [44] recently reported that the intermediate host snails *O. hupensis robertsoni* in Sichuan and the snail *O. hupensis hupensis* in the ML reaches had a 10.3% genetic distance, strongly indicating that the two subspecies may differ at the species level. In a phylogenetic study on the Schistosomatidae, Lockyer et al. [45] considered that schistosomes in east Asia and their intermediate hosts in the Pomatiopsidae may be considered as the only co-evolutionary model between schistosomes and their intermediate host snails. Davis et al. [46] also speculated, as snail population forms have diverged genetically, so must their associated schistosomes or else become regionally extinct. However, it would be only possible to examine such relationship if the intermediate host snails and schistosomes are collected from a large geographical range in east Asia.

In a very small-scale area in Anhui province of China, Rudge et al. [40] detected strong genetic differentiation in *S. japonicum* between two types of habitats, lake/marshland region and hilly region, and suggested that contrasting host reservoirs may be associated with the genetic differentiation, with rodents and dogs being important infection reservoirs in hilly regions and bovines in lake/marshland regions. On the other hand, they found little or no parasite genetic differentiation among host species within most villages; but in another study, Wang et al. [47] reported that schistosomes were separated into two clades representing the parasites from different definitive hosts. It seems likely that *S. japonicum* has undergone genetic differentiation in a relatively small-scale area, as in a large geographical region reported in this study. In the above two studies, miracidia from definitive hosts were examined with microsatellite markers. In the present study, adult parasites were obtained through infecting mice with cercariae. As definitive host-based genetic variation in *S. japonicum* has been noticed [40], [47], the selection pressure through definitive host may need to be further investigated.

Unexpectedly, three haplotypes representing some schistosomes from three localities, each in Hubei, Hunan, Anhui provinces, were actually clustered together within another clade containing all haplotypes from Sichuan and Yunnan provinces. It is, however, at present impossible to explain this mixed cluster. As the movement of people has been frequent in China [48], the possible transmission through definitive host cannot be ruled out as a possible interpretation.

In conclusion, substantial genetic diversity was demonstrated in populations of *S. japonicum* in schistosomiasis endemic areas in mainland China. Overall, a significant correlation was observed between the genetic distance and the geographical distance among the populations. It is apparent that the populations from Sichuan and Yunnan provinces in SW China exhibited a relatively low level of genetic variation, and were genetically different from the populations in the ML reaches of the Yangtze River, which had a much more complicated genetic diversity. Such obvious genetic diversity should be taken into consideration in guiding any strategic control programmes and/or vaccine development/trials in the future.

## Supporting Information

Table S1 **Within-locality diversity of** ***Schistosoma japonicum*** **from 18 localities in mainland China based on Cytb-ND4L-ND4 fragment.**
(XLS)

Table S2 **Within-locality diversity of** ***Schistosoma japonicum*** **from 18 localities in mainland China based on ND1 fragment.**
(XLS)

Table S3 **Within-locality diversity of** ***Schistosoma japonicum*** **from 18 localities in mainland China based on 16S-12S fragment.**
(XLS)

Table S4 **Geographical population pairwise genetic distance (** ***F*** **st) and gene flow (Nm) based on ND1.**
(XLS)

Table S5 **Geographical population pairwise genetic distance (** ***F*** **st) and gene flow (Nm) based on 16S-12S.**
(XLS)

Table S6 **Geographical population pairwise genetic distance (** ***F*** **st) and gene flow (Nm) based on Cytb-ND4L-ND4.**
(XLS)
